# Associations of phosphorus concentrations with medial arterial calcification in lower-extremity arteries and diabetic foot in people with diabetes: a retrospective cross-sectional study

**DOI:** 10.1186/s12933-024-02361-5

**Published:** 2024-07-25

**Authors:** Peishan Li, Qingxian Li, Mingyu Tang, Xingyun Hu, Jing Tian, Jianbin Zhang, Chuan Yang, Baile Zhu

**Affiliations:** 1 Sun Yat-Sen Memorial Hospital, Sun Yat-Sen University, Guangzhou, 510120 China; 2 Shenzhen Longhua District Central Hospital, Shenzhen, 518110 China; 3 Zhongshan People’s Hospital, Zhongshan, 528403 China

**Keywords:** Phosphorus, Medial arterial calcification, Diabetic foot

## Abstract

**Background:**

The aim of this study was to investigate the associations of blood phosphorus levels with the risk of developing medial arterial calcification (MAC) in lower-limb arteries and diabetic foot (DF) in diabetes patients. We sought to enhance the understanding of the pathophysiology of diabetic complications and develop strategies to mitigate diabetes-related risks.

**Methods:**

We conducted a retrospective analysis of 701 diabetic patients from the Department of Endocrinology at Sun Yat-Sen Memorial Hospital (2019–2023). We utilized multimodel-adjusted logistic regression to investigate the associations of serum phosphorus levels and the risk of developing MAC and DF. Restricted cubic spline plots were employed to model the relationships, and threshold analysis was used to identify inflection points. Subgroup analyses were performed to explore variations across different demographics. The diagnostic utility of phosphorus concentrations was assessed via the C index, net reclassification improvement (NRI), and integrated discrimination improvement (IDI).

**Results:**

Of the 701 patients (mean age 63.9 years; 401 (57.20%) were male), 333 (47.50%) had MAC, and 329 (46.93%) had DF. After controlling for numerous confounding variables, each one-unit increase in phosphorus concentrations was associated with an increased risk of developing MAC (OR 2.65, 95% CI 1.97–3.57, p < 0.001) and DF (OR 1.54, 95% CI 1.09–2.18, p = 0.014). Phosphorus levels demonstrated a linear risk association, with risk not being uniform on either side of the inflection point, which was approximately 3.28 mg/dL for MAC and varied for DF (3.26 to 3.81 mg/dL). Adding the phosphorus as an independent component to the diagnostic model for MAC and DF increased the C index, NRI, and IDI to varying degrees.

**Conclusions:**

Elevated serum phosphorus levels are significantly associated with an increased risk of developing MAC and DF among diabetic people. These findings suggest that phosphorus management could be integrated into routine diagnostic processes to improve the identification and management of lower-extremity diabetic complications.

**Graphical abstract:**

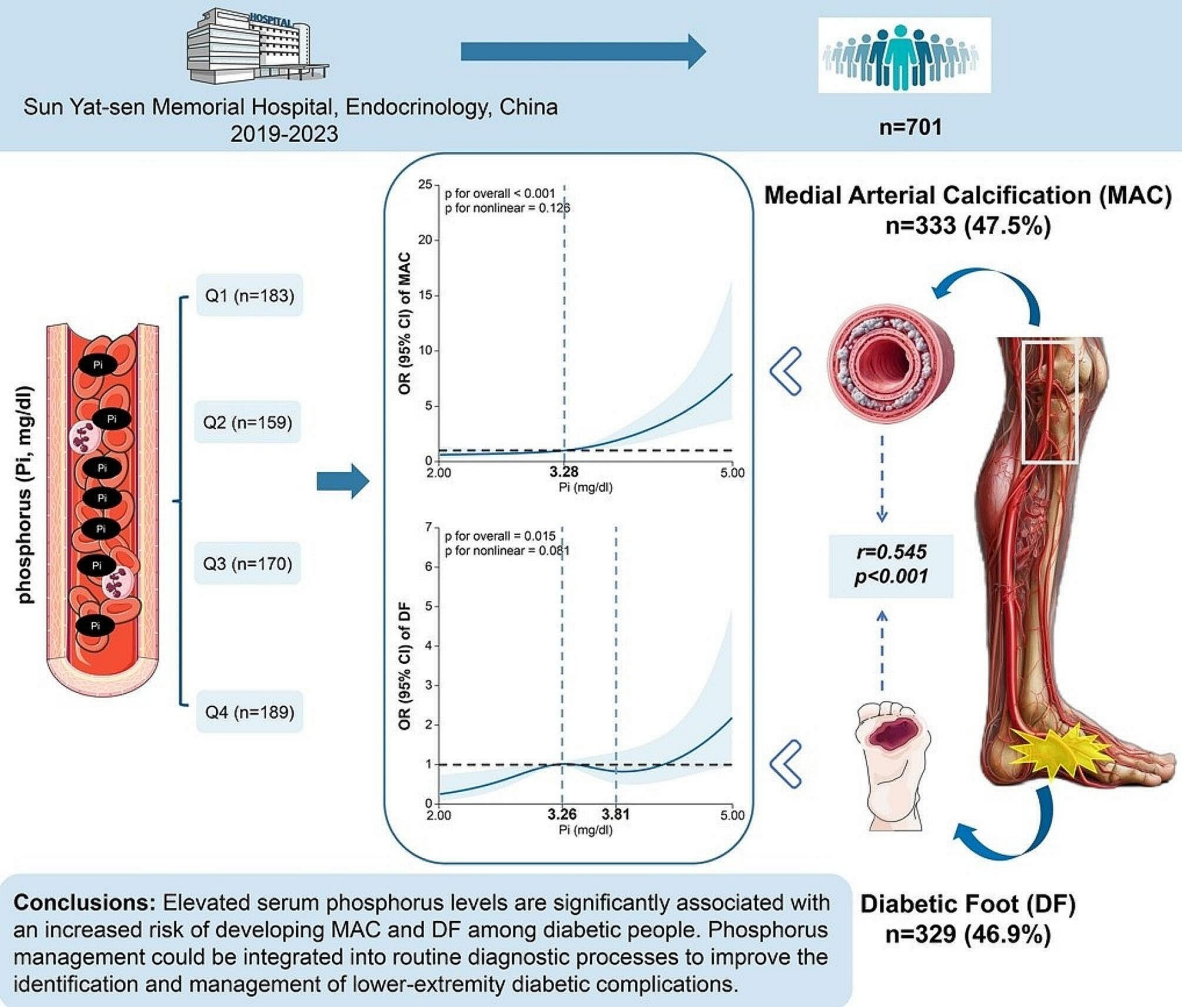

**Supplementary Information:**

The online version contains supplementary material available at 10.1186/s12933-024-02361-5.

## Introduction

Recent studies have linked phosphorus levels to cardiovascular disease risk, particularly by stimulating blood vessel calcification, which independently predicts cardiovascular mortality. This association is notably harmful in dialysis patients [1–4]. Medial arterial calcification (MAC), a condition distinct from atherosclerosis, is characterized by calcium and phosphorus deposition in the media of blood vessels and frequently occurs in individuals with diabetes, end-stage renal disease (ESRD), chronic kidney disease (CKD), and advanced age. Owing to its high prevalence in the lower-limb arteries, MAC contributes to chronic limb ischemia and diabetic foot (DF) [5–8].

Sadiq Ahmed et al. reported a link between increased phosphorus levels and vascular calcification in uremia patients, with histologic studies confirming calcification in the media layer of vascular smooth muscle cells [9]. Elevated phosphorus concentrations are also associated with an increased risk of experiencing cardiovascular events in individuals without preexisting cardiovascular disease, particularly in individuals with younger ages and those with normal kidney function [4, 10, 11]. For example, studies by Marcello and Ravi Dhingra showed that serum phosphorus levels were linked to all-cause mortality, new heart failure, and coronary events, even in populations with normal serum phosphorus levels [12]. The correlation between elevated phosphorus and mortality is more pronounced in individuals with diabetes [1].

However, the relationship between phosphorus concentrations and vascular calcification in patients with diabetes remains inconsistent and varies with disease type, ethnicity, and study location. The connection between phosphorus concentrations and lower-extremity arterial MAC has not been thoroughly investigated in diabetes patients who do not require dialysis. High phosphorus levels are linked to foot ulcers in uremia patients [13]. Current research focused on phosphorus in DF is limited, and its potential as a risk factor deserves attention because of its known associations with bone health and kidney function. DF significantly increases the risk of mortality and other severe adverse outcomes in patients with type 2 diabetes (T2DM), which requires substantial attention and early intervention [14].

Therefore, it is crucial to investigate the relationships of phosphorus with the risk of developing lower-extremity MAC and DF in diabetes patients and to evaluate the blood phosphorus as an independent diagnostic marker. In this research, we aimed to elucidate the connection between phosphorus levels and DF pathology, potentially leading to more precise and effective prevention and treatment protocols.

## Methods

### Study design and participants

This retrospective study included patients with T2DM from the Endocrinology Department of Sun Yat-sen Memorial Hospital between 2019 and 2023. The study population included individuals over 18 years of age, without restrictions on sex or ethnicity, who had renal function classified as CKD stage 1–4. Exclusion criteria included those with type 1 diabetes, gestational diabetes, and other specific types of diabetes as classified by the American Diabetes Association; patients undergoing renal replacement therapy (including hemodialysis, peritoneal dialysis, and kidney transplantation); individuals with severe hematologic diseases (such as leukemia, lymphoma, and severe anemia); cancerous tumors; recent cardiovascular or cerebrovascular events (within the last 3 months); long-term use of hormones or immunosuppressants; pregnant or breastfeeding women; and those with serious mental or infectious diseases. The final study sample consisted of 701 participants (Additional File 1: Fig. S1). This study was conducted in strict adherence to the Strengthening the Reporting of Observational Studies in Epidemiology (STROBE) guidelines (https://www.strobe-statement.org).

### Data collection and definitions

The primary medical history of the patients was collected through a case system at Sun Yat-Sen Memorial Hospital, Sun Yat-Sen University, and general case information, such as the patient’s sex, age, body mass index (BMI), history of hypertension (HBP), history of coronary heart disease (CHD), and history of diabetes-related complications, was collected. The diagnosis of diabetes was made according to the 1999 World Health Organization criteria [15]. The patients included in the study had their blood collected early in the morning of the day after admission after fasting for 12 h. The blood samples were sent to the laboratory department of our hospital for testing. Albumin (ALB, g/L), uric acid (UA, μmol/L), creatinine (SCr, μmol/L), phosphorus (Pi, mg/dL), Ca, corrected calcium (Ca, mg/dL), total serum cholesterol (TC, mmol/L), triglycerides (TG, mmol/L), high-density lipoprotein (HDL, mmol/L), low-density lipoprotein (LDL-C, mmol/L), high-sensitivity C-reactive protein (hs-CRP, mg/L), superoxide dismutase (SOD, U/ml), glycosylated hemoglobin (HbA1c, %), and fasting plasma glucose (FPG, mmol/L) were measured. The estimated glomerular filtration rate (eGFR) was calculated via the 2009 CKD-EPI formula, which was used to categorize CKD stages 1 to 4 [16]. In addition, we used the lipid composite index (LCI) to represent the overall lipid profile. LCI = TC × TG × LDL-C/HDL-C. The triglyceride‒glucose (TyG) index represents insulin resistance [17]. TyG = ln (fasting TG (mg/dL) × fasting glucose (mg/dL)/2). If ALB < 40 g/L, corrected calcium (mg/dL) = total calcium (mg/dL) + 0.8 × (4-ALB (g/dL)).

Two specialized physicians collected and verified the above data. The data collected were approved by the Medical Ethics Committee of Sun Yat-Sen Memorial Hospital under license No. SYSKY-2023-418-01.

### Study outcomes

Our institution boasts a strong vascular ultrasound team, and all ultrasounds are interpreted by professors with extensive experience, ensuring the readability and reliability of the results. A LOGIQ E9 machine (GE, USA) equipped with a 9L or 5–12 MHz probe was used for cardiovascular ultrasound evaluation of the lower extremities. On the basis of the expertise of our hospital’s vascular ultrasound team, the external iliac, superficial femoral, popliteal, anterior tibial, posterior tibial, peroneal, and dorsalis pedis arteries were scanned in both the transverse and longitudinal planes to assess calcification. MAC was defined by the presence of smooth, linear, nonstenotic, and strongly echogenic bands [18]. For the diagnosis of DF, we based our diagnosis on an examination of the collected history, a physical examination, and the International Working Group (IWG)-DF guidelines. The diagnosis was characterized by profound tissue destruction, infection, or ulceration of the foot [19].

### Statistical analysis

The participants were categorized into four groups based on phosphorus quartiles (25th, 50th, and 75th percentiles). Continuous data with a normal distribution are presented as the mean (standard deviation (SD)), and skewed distribution data are presented as the median (interquartile range (IRQ)). Categorical data are presented as frequencies and percentages (%). Group differences in continuous variables were evaluated via either ANOVA or the Kruskal‒Wallis test, whereas categorical variables were analyzed via the chi-square test or the Cochran‒Mantel‒Haenszel (CMH) test. For the comparison of continuous variables between two groups, *t* tests (for normally distributed data) or rank-sum tests (for nonnormally distributed data) were used.

We used logistic regression models to calculate odds ratios (ORs) and 95% confidence intervals (CIs) for the associations between phosphorus levels and the occurrence of MAC and DF. Four models were created: one univariate model and three multivariate models. The selection of confounders was based on clinical expertise. The unadjusted model did not account for any variables. Model 1 was adjusted for calcium concentrations. Model 2 was further adjusted for sex, age, HBP history, diabetic neuropathy status, the eGFR, and HbA1c. Model 3 was further adjusted for ALB, UA, LDL-C, TC, TG, HDL-C, SOD, and hs-CRP concentrations. Phosphorus concentrations were examined independently as both a continuous variable and a categorical variable. The model fitted by Model 3 was used to predict the risk of developing MAC and DF. Scatter plots were constructed, and correlation analysis was performed. Furthermore, we examined and visualized the linear correlations of phosphorus concentrations with the risk of developing MAC and DF via restricted cubic splines (RCSs). Using the likelihood ratio test, we evaluated the pattern of risk alteration prior to and after the inflection point by applying threshold analysis. The nodes were chosen explicitly at the 5th, 35th, 65th, and 95th percentiles, with the inflection point as the reference. The subgroup analyses were categorized according to age, sex, CKD stages (CKD 1–2, CKD 3–4), presence of diabetic neuropathy, history of HBP, calcium, HbA1c, albumin, TyG index, hs-CRP, and LCI levels. The stratification foundation mentioned above was accounted for together with the sample size of this study and the mean or median of each variable. Additionally, interaction analysis was conducted via the multiplicative intersection approach. To further ensure the robustness of our results, we conducted three sensitivity analyses. First, we included CHD as a covariate in Model 3. Second, we replaced the eGFR with SCr concentrations in the model. Third, we used propensity score matching (PSM) to minimize the influence of confounding factors. Each treated individual was matched with a control individual with the closest propensity score via 1:1 nearest neighbor matching. We then repeated the analysis of the relationships of phosphorus concentrations with the risk of developing MAC and DF, confirming the reliability of our findings.

In addition, we fitted a diagnostic model including sex, age, HBP, neuropathy, eGFR, HbA1c, ALB, UA, LDL-C, TC, TG, HDL-C, SOD, hs-CRP, and Ca, and then built an enhanced model that incorporated these variables along with phosphorus. We used Harrell’s concordance statistic (C index), the integrated discriminant index (IDI), and the net reclassification index (NRI) to find out how phosphorus improved MAC and DF diagnosis. Using the same methodology, we analyzed the incremental diagnostic value of calcium and the calcium-phosphorus product for identifying MAC and DF.

Statistical analyses were performed via R software (version 4.3.3; R Core Team, 2024; R Foundation for Statistical Computing, Vienna, Austria). Forest plots, violin plots, percentage bar graphs, and RCS plots were generated via the “forestploter”, “gghalves”, “ggplot2”, and “rcsplot” packages. NRI and IDI calculations were performed via the “PredictABEL” package, and mediation analysis was conducted via the “mediation” package. A two-sided p < 0.05 was considered to indicate statistical significance.

## Results

### Baseline characteristics

**Fig. 1:**
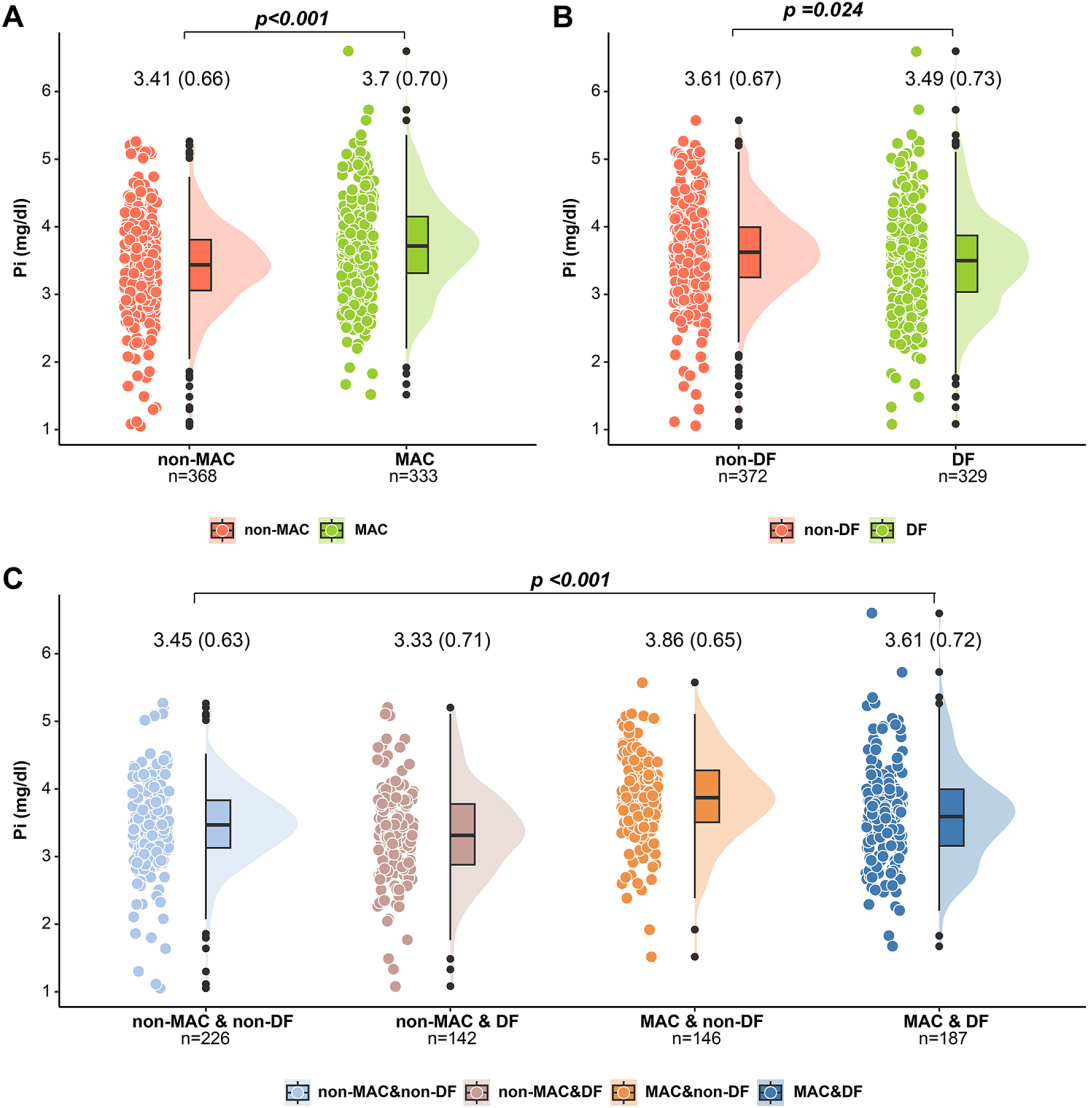
Differences in blood phosphorus levels between MAC and DF patients. *mean (SD).MAC* medial arterial calcification, *DF* diabetic foot, *Pi* phosphorus (mg/dL)

Blood phosphorus concentrations were divided into four quartiles: Q1 [2.11 (1.05, 3.16)], Q2 [3.36 (3.16, 3.56)], Q3 [3.75 (3.56, 3.93)], and Q4 [5.26 (3.93, 6.60)]. The baseline characteristics of each quartile group are presented in Table 1. The mean age of the population included in the study was 63.9 years, with a majority (445, 63.48%) older than 60 years. There were 300 females (42.80%) and 401 males (57.20%). There were 333 individuals (47.5%) with MAC and 329 individuals (46.93%) with DF. The mean total phosphorus was 3.55 mg/dL. Moreover, the percentages of patients with MAC and DF; the CKD stage; ALB concentrations; the calcium‒phosphorus product; and SCr, UA, CHOL, LDL‒C, HDL‒C, hs‒CRP, and SOD concentrations differed significantly among the different quartiles of phosphorus (p < 0.05; Table 1). However, the percentages of patients with HBP history and diabetic nephropathy status; TG concentrations; and the LCI and TyG index were not significantly different among the different phosphorus concentration quartiles (p > 0.05; Table 1). In the violin plots (Fig. 1), participants with MAC had greater phosphorus levels than non-MAC participants did (3.72 vs. 3.41, p < 0.001; Fig. 1A). Participants with DF had lower phosphorus levels than non-MAC participants did (3.49 vs. 3.61, p = 0.025; Fig. 1B). There was a statistically significant difference in phosphorus levels between the non-MAC&non-DF, non-MAC&DF, MAC&non-DF, and MAC&DF groups (p < 0.001; Fig. 1C). In addition, other differences between the MAC group and non-MAC, DF, and non-DF groups were compared (Additional File 1: Table S1).

**Table 1:** Clinical characteristics of the subjects (n = 701)

	Total (n = 701)	Phosphorus (mg/dL)				
		Q1 (n = 183)	Q2 (n = 159)	Q3 (n = 170)	Q4 (n = 189)	p Value
Clinical materials						
MAC (%)						< 0.001
No	368 (52.50)	113 (61.75)	104 (65.41)	82 (48.2)	69 (36.51)	
Yes	333 (47.50)	70 (38.25)	55 (34.59)	88 (51.76)	120 (63.49)	
Age	63.90 (13.29)	66.49 (14.24)	64.25 (13.58)	63.37 (12.57)	61.57 (12.35)	0.004
< 60 years (%)	256 (36.52)	50 (27.32)	56 (35.22)	68 (40.00)	82 (43.39)	0.001
> 60 years (%)	445 (63.48)	133 (72.68)	103 (64.78)	102 (60.00)	107 (56.61)	
Sex						0.116
Female (%)	300 (42.80)	70 (38.25)	63 (39.62)	73 (42.94)	94 (49.74)	
Male (%)	401 (57.20)	113 (61.75)	96 (60.38)	97 (57.06)	95 (50.26)	
BMI (kg/m^2^)	23.85 (3.85)	23.49 (3.84)	23.89 (3.58)	23.94 (3.95)	24.07 (4.01)	0.512
SBP (mmHg)	136.03 (21.58)	136.79 (21.92)	138.91 (22.37)	135.09 (22.27)	133.72 (19.72)	0.137
DBP (mmHg)	74.35 (11.18)	74.45 (11.74)	75.88 (11.39)	74.66 (10.42)	72.70 (10.99)	0.065
Diabetes foot (%)						0.036
No	372 (53.07)	82 (44.81)	86 (54.09)	91 (53.53)	113 (59.79)	
Yes	329 (46.93)	101 (55.19)	73 (45.91)	79 (46.47)	76 (40.21)	
Hypertension (%)						0.851
No	271 (38.66)	74 (40.44)	57 (35.85)	66 (38.82)	74 (39.15)	
Yes	430 (61.34)	109 (59.56)	102 (64.15)	104 (61.18)	115 (60.85)	
Nephropathy (%)						0.118
No	379 (54.07)	86 (47.99)	91 (57.23)	100 (58.82)	102 (53.97)	
Yes	322 (45.93)	97 (53.01)	68 (42.77)	70 (41.18)	87 (46.03)	
Neuropathy (%)						0.437
No	223 (31.81)	60 (32.79)	56 (35.22)	46 (27.06)	61 (32.28)	
Yes	478 (68.19)	123 (67.21)	103 (64.78)	124 (72.94)	128 (67.72)	
CHD (%)						0.022
No	544 (77.60%)	134 (73.22%)	115 (72.33%)	143 (84.12%)	152 (80.42%)	
Yes	157 (22.40%)	49 (26.78%)	44 (27.67%)	27 (15.88%)	37 (19.58%)	
Laboratory examination						
Ca (mg/dL)	9.82 (0.73)	9.90 (0.84)	9.74 (0.72)	9.85 (0.67)	9.80 (0.66)	0.197
Pi (mg/dL)	3.55 (0.70)	2.71 (0.44)	3.37 (0.11)	3.73 (0.11)	4.38 (0.42)	< 0.001
Ca–Pi product (mg/dL)^2^	33.42 (7.08)	25.03 (4.50)	31.61 (1.65)	35.10 (1.62)	41.57 (4.65)	< 0.001
ALB (g/L)	34.48 (6.95)	31.62 (7.51)	35.59 (6.74)	34.64 (6.86)	36.16 (5.72)	< 0.001
Scr (μmol/L)	82.00 (36.00)	81.00 (32.00)	82.00 (33.50)	78.50 (37.00)	85.00 (51.00)	< 0.001
eGFR (mL/min/1.73 m^2^)	74.8 (25.7)	76.4 (24.0)	75.6 (23.9)	76.9 (23.9)	70.7 (29.8)	0.075
CKD stage (%)						0.009
CKD 1	226 (32.24)	62 (33.88)	49 (30.82)	61 (35.88)	54 (28.57)	
CKD 2	271 (38.66)	70 (38.25)	66 (41.51)	68 (40.00)	67 (35.45)	
CKD 3	170 (24.25)	46 (25.14)	41 (25.79)	35 (20.59)	48 (25.40)	
CKD 4	34 (4.85)	5 (2.73)	3 (1.89)	6 (3.53)	20 (10.58)	
UA (μmol/L)	342.55 (118.73)	297.99 (117.37)	350.24 (116.28)	342.16 (117.06)	379.58 (110.05)	< 0.001
FPG (mmol/L)	7.21 (2.70)	7.61 (3.11)	7.28 (2.68)	7.01 (2.54)	6.93 (2.39)	0.071
HbA1c (%)	8.78 (2.34)	9.13 (2.50)	8.63 (2.24)	8.62 (2.24)	8.73 (2.32)	0.129
CHOL (mmol/L)	4.46 (1.42)	4.07 (1.36)	4.65 (1.41)	4.46 (1.38)	4.68 (1.46)	< 0.001
TG (mmol/L)	1.55 (1.35)	1.60 (1.49)	1.45 (1.01)	1.38 (0.96)	1.71 (1.70)	0.090
LDL-C (mmol/L)	2.80 (1.00)	2.57 (0.95)	2.89 (1.02)	2.79 (0.98)	2.96 (1.03)	0.001
HDL (mmol/L)	1.04 (0.40)	0.92 (0.48)	1.10 (0.38)	1.08 (0.38)	1.05 (0.33)	< 0.001
hs-CRP (mg/L)	4.91 (35.21)	20.37 (67.63)	3.69 (33.74)	4.12 (29.72)	2.56 (13.84)	< 0.001
LCI	14.66 (21.56)	14.72 (19.86)	14.76 (19.71)	11.74 (19.53)	17.04 (23.54)	0.202
TyG index	8.85 (0.69)	8.91 (0.75)	8.84 (0.64)	8.74 (0.67)	8.92 (0.67)	0.073
SOD (U/mL)	143.26 (34.16)	133.89 (27.34)	149.91 (39.15)	143.77 (31.14)	146.29 (36.45)	< 0.001

Phosphorus (mg/dL): Q1 [2.11 (1.05,3.16)], Q2 [3.36 (3.16,3.56)], Q3 [3.75 (3.56,3.93)], Q4 [5.26 (3.93, 6.60)]

The data are presented as the means (SDs), numbers (percentages), or medians (IQRs). *MAC* medial arterial calcification, *DF* diabetic foot, *CHD* coronary heart disease, *Pi* phosphorus, *Ca* corrected calcium, *Ca × Pi* calcium‒phosphorus product, *BMI* body mass index, *ALB* albumin, *SCr* serum creatinine, *eGFR* estimated glomerular filtration rate, *UA* uric acid, *HbA1c* blood glycosylated hemoglobin, *FPG* fasting plasma glucose, *CHOL* total cholesterol, *TG* triglyceride, *LDL-C* low-density lipoprotein cholesterol, *HDL* high-density lipoprotein cholesterol, *LCI* lipid comprehensive index, *hs-CRP* high-sensitivity C-reactive protein, *SOD* superoxide dismutase, *TyG index* triglyceride–glucose index

### Relationships of phosphorus concentrations with the risk of developing MAC and DF

**Fig. 2:**
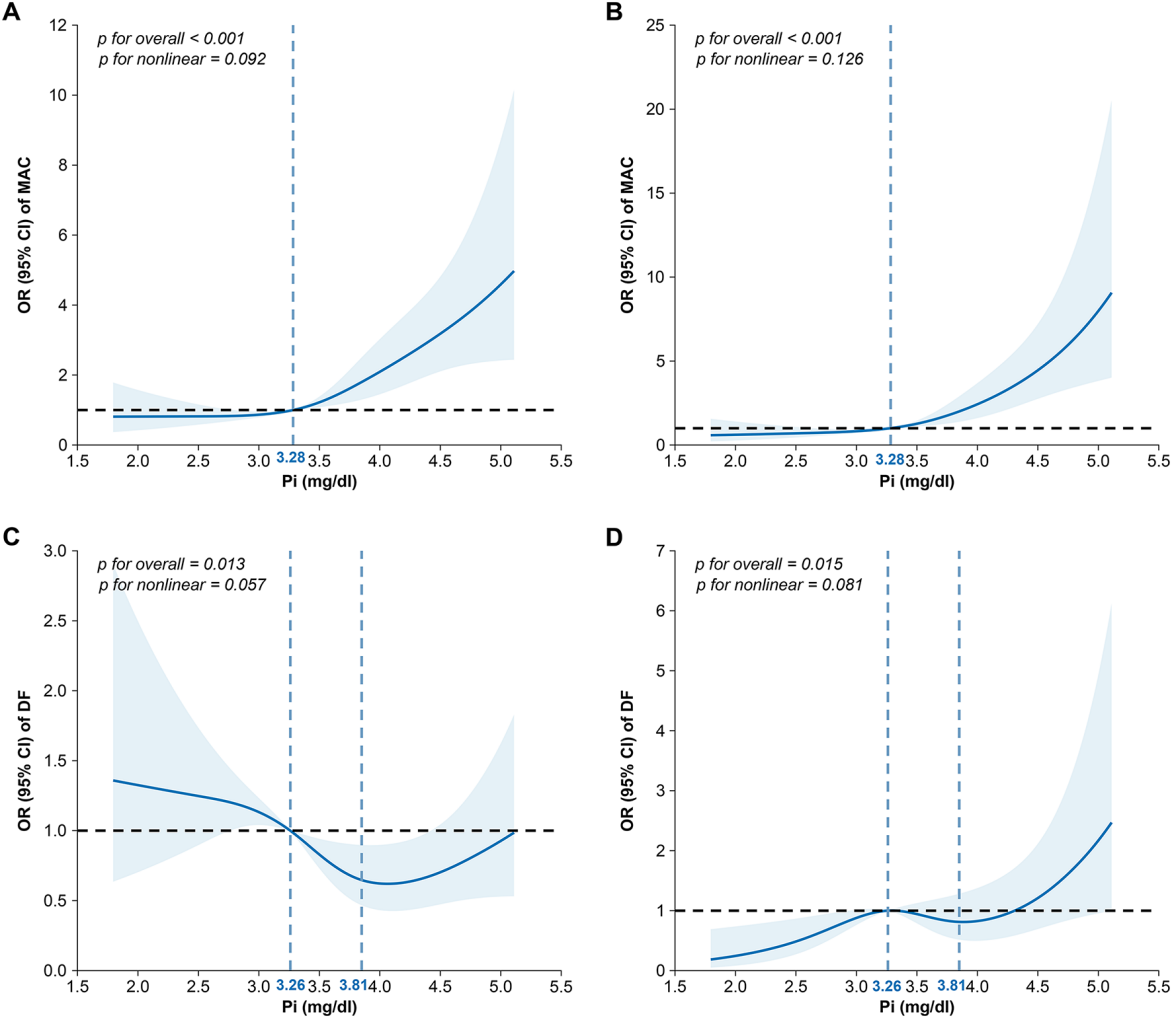
RCS plots of the risk of developing MAC (**A** and **B**) and DF (**C** and **D**) by phosphorus content. Univariate analysis of the risk of developing MAC (**A**) and DF (**C**) according to phosphorus concentrations. Multifactorial analysis for the risk of developing MAC (**B**) and DF (**D**) according to phosphorus concentrations adjusted by sex, age, HBP history, neuropathy, Ca, eGFR, HbA1c, ALB, UA, LDL-C, TC, TG, HDL-C, SOD, and hs-CRP. Data were fitted by a logistic regression model, and the model was constructed with 4 knots at the 5th, 35th, 65th, and 95th percentiles of phosphorus (reference is the threshold in Table 3). The solid lines indicate ORs, and the shadow shapes indicate 95% CIs. *OR* odds ratio, *CI* confidence interval, *DF* diabetic foot, *MAC* medial arterial calcification

The ORs for the occurrence of MAC and DF on the basis of phosphorus in the univariate and multivariate logistic regression models, with multiple corrections for phosphorus concentrations, are shown in Table 2. The risk of developing MAC increased with each unit increase in phosphorus in any model, whether due to one or multiple factors. Similarly, when phosphorus was put into quartiles (using quartile 2 as a reference), the risk of developing MAC increased as the percentiles of phosphorus increased (all p < 0.001; Table 2). The fourth quartile of phosphorus exhibited the strongest association with the risk of developing MAC (OR 3.96, 95% CI 2.43–6.46; p < 0.001; Table 2). The above trend was also reflected in the multivariate-adjusted RCS (Fig. 2A, B), with phosphorus concentrations in the range of 1.5 to 5.5 mg/dL and the MAC risk showing an increasing trend with increasing phosphorus. Combining the graphical trend and threshold analysis (Table 3), we identified one inflection point of the curve at 3.28 mg/dL. The OR was 1.53 (95% CI 0.83–2.92) before the inflection point and 3.56 (95% CI 2.37–5.45) after it, of which the strongest correlation was with phosphorus > 3.28 mg/dL (Table 3).

**Table 2:** Multifactor logistic regression model of the associations between phosphorus and the risk of developing MAC & DF

All patients (n = 701)	Event/N	Unadjusted		Model 1		Model 2		Model 3	
		OR (95% CI)	p-value	OR (95% CI)	p-value	OR (95% CI)	p-value	OR (95% CI)	p-value
Continuous (per unit)	333/701	1.99 (1.57–2.52)	< 0.001	2.09 (1.64–2.66)	< 0.001	2.27 (1.74–2.97)	< 0.001	2.65 (1.97–3.57)	< 0.001
Quartiles									
Q1	55/159	1.17 (0.75–1.82)	0.483	1.22 (0.78–1.91)	0.764	1.10 (0.69–1.77)	0.690	0.96 (0.59–1.56)	0.884
Q2	70/183	Ref.	–	Ref.	–	Ref.	–	Ref.	–
Q3	88/170	2.03 (1.30–3.16)	0.002	2.01 (1.29–3.14)	0.003	2.11 (1.32–3.99)	0.002	2.12 (1.32–3.42)	0.002
Q4	120/189	3.29 (2.12–5.11)	< 0.001	3.20 (2.05–4.98)	< 0.001	3.85 (2.39–6.22)	< 0.001	3.96 (2.43–6.46)	< 0.001
p for trend		< 0.001		< 0.001		< 0.001		< 0.001	
Continuous (per unit)	329/701	0.78 (0.63–0.97)	0.024	0.79 (0.62–1.01)	0.060	0.80 (0.61–1.04)	0.089	1.54 (1.09–2.18)	0.014
Quartiles									
Q1	101/183	1.45 (0.95–2.22)	0.087	1.19 (0.74–1.93)	0.476	1.27 (0.76–2.13)	0.359	0.53 (0.29–0.99)	0.048
Q2	73/159	Ref.	–	Ref.	–	Ref.	–	Ref.	–
Q3	79/170	1.02 (0.66–1.58)	0.919	0.89 (0.55–1.45)	0.642	0.90 (0.54–1.52)	0.706	0.93 (0.52–1.66)	0.813
Q4	76/189	0.79 (0.52–1.21)	0.285	0.70 (0.43–1.12)	0.136	0.75 (0.45–1.26)	0.283	0.98 (0.55–1.73)	0.932
p for trend		0.095		0.058		0.160		0.687	

Phosphorus (mg/dL): Q1 [2.11 (1.05,3.16)], Q2 [3.36 (3.16,3.56)], Q3 [3.75 (3.56,3.93)], and Q4 [5.26 (3.93, 6.60)]. All of the models use Q2 as the reference

Model 1, adjusted by Ca

Model 2, Adjusted by Model 1 + sex, age, HBP history, neuropathy, eGFR, and HbA1c

Model 3, Adjusted by Model 2 + ALB, UA, LDL-C, TC, TG, HDL-C, SOD, hs-CRP

**Table 3:** Threshold analysis of phosphorus on MAC and DF

	Event/N	Adjusted OR (95% CI)	p-Value
Medial arterial calcification (MAC)			
Total		2.73 (2.04–3.67)	< 0.001
Fitting by two-piecewise linear model			
Inflection point		3.28	
Pi < 3.28	80/213	1.53 (0.83–2.92)	0.180
Pi ≥ 3.28	253/488	3.56 (2.37–5.45)	< 0.001
p for Log-likelihood ratio		0.053	
Diabetes foot (DF)			
Total		1.54 (1.09–2.18)	0.014
Fitting by three-piecewise linear model			
Inflection point		3.26, 3.81	
Pi < 3.26	118/213	3.24 (1.01–10.39)	0.048
3.26 ≤ Pi < 3.81	117/257	1.39 (0.17–11.10)	0.754
Pi ≥ 3.81	94/131	3.68 (1.39–9.72)	0.008
p for Log-likelihood ratio		0.035	

Adjusted for sex, age, HBP, Neuropathy, eGFR, HbA1c, ALB, UA, LDL-C, TC, TG, HDL-C, SOD, hs-CRP, Ca

The relationship between phosphorus concentrations and the risk of developing DF appears to be more complex. The unadjusted model revealed that the risk of developing DF decreased by 22% for every unit increase in phosphorus (OR 0.78, 95% CI 0.63–0.97; p < 0.05; Table 2). However, in Model 3, the risk increased by 57% (OR 1.57, 95% CI 1.12–2.22, p < 0.05; Table 2) for each 1-unit increase in phosphorus. On the other hand, when participants were categorized into quartiles (using Q2 as a reference), those in the lowest percentile for phosphorus were less likely to develop DF (OR 0.53, 95% CI 0.29–0.99, p = 0.048; Table 2). Moreover, the risk was essentially indistinguishable in the other percentile groups. Although the linear relationship was less pronounced than that of MAC, the relationship between phosphorus concentrations and the risk of developing DF also showed a significant positive linear trend (p for overall < 0.05; Fig. 2C, D). The trend of DF risk varies with different ranges of phosphorus concentrations. Three regressions revealed two inflection points of the curve: 3.26 and 3.81 mg/dL, with ORs and 95% CIs of 3.24 (1.01–10.39), 1.39 (0.17–11.10), and 3.68 (1.39–9.72), with a stronger association observed for phosphorus levels < 3.26 and ≥ 3.81 mg/dL (Table 3).

**Fig. 3:**
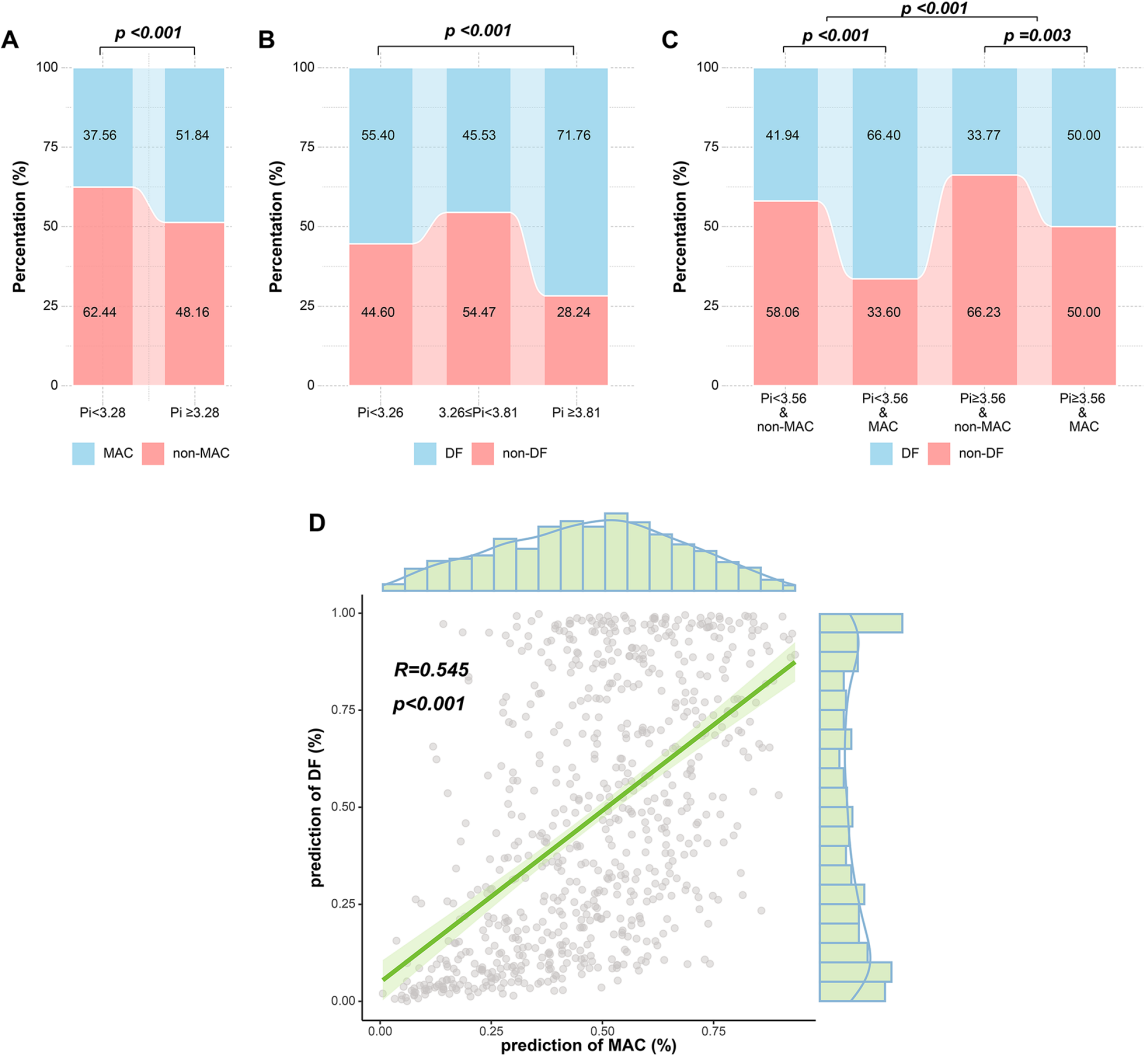
MAC and DF proportions in various phosphorus (**A**–**C**) and correlations (**D**). Proportion of MAC with different phosphorus (**A**). Proportion of DF in different blood phosphorus (**B**). 3.28, 3.26 and 3.81 mg/dL were the result of threshold effect analysis in Table 2. Proportion of DF in different blood phosphorus and MAC (C). 3.56 mg/dL is median for Pi. Correlation between predicted probability of MAC and DF risk (**D**). The prediction is calculated by Model 3. *DF* diabetes foot, *MAC* medial arterial calcification. p < 0.001 by chi-square (**A**, **B**) and CMH tests (**C**)

The percentages of patients with MAC and DF at different phosphorus thresholds were also compared. Increased phosphorus levels were associated with greater proportions of patients with MAC and DF (p < 0.001; Fig. 3A, B). Additionally, those with MAC had a greater incidence of DF, regardless of phosphorus levels below the median (3.56 mg/dL) (p < 0.001; Fig. 3C). Additionally, MAC and DF probabilities were estimated independently for each patient via Model 3 and presented as a scatter plot, indicating a substantial linear association (r = 0.545, p < 0.001; Fig. 3D). Finally, for Model 3, we also constructed separate multivariate regression models for the outcomes of MAC and DF (Additional File 1: Table S2). The results indicated that, in addition to phosphorus, age, neuropathy, and HbA1c were significantly associated with the risk of developing MAC and DF (p < 0.05).

Additionally, mediation analysis indicated that the total effect and direct effect of phosphorus concentrations on the risk of developing DF were significant (p < 0.05), whereas the mediating effect on the risk of developing MAC was not significant (p > 0.05) (Additional File 1: Fig. S2).

**Fig. 4:**
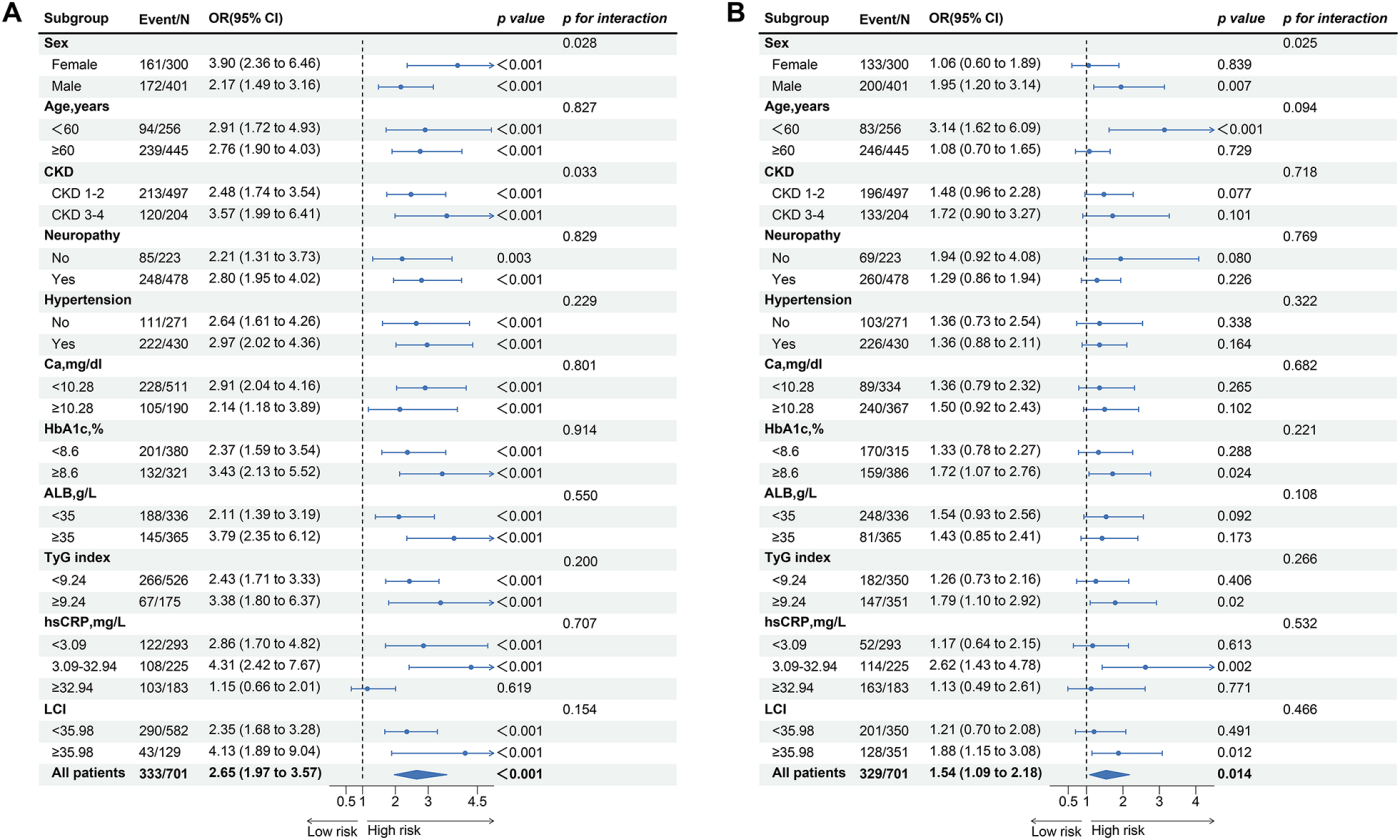
Subgroup analysis of phosphorus with MAC (**A**) and DF (**B**). Adjusted by sex, age, HBP, Neuropathy, eGFR, HbA1c, ALB, UA, LDL-C, TC, TG, HDL-C, SOD, hs-CRP, Ca

**Fig. 5:**
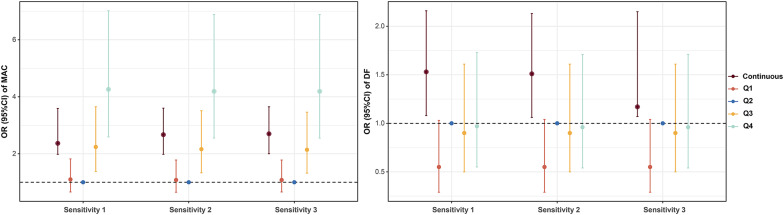
Sensitivity Analysis of Phosphorus in Multivariate Adjustment. Sensitivity analysis 1: Incorporating status CHD into Model 3. Sensitivity analysis 2: Replacing the eGFR with SCr in Model 3. Phosphorus concentrations were analyzed both as a continuous variable and as a categorical (quartiles) variable. Sensitivity analysis 3: PSM. *DF* diabetes foot, *MAC* medial arterial calcification

Further subgroup analysis is shown in Fig. 4. A positive correlation between phosphorus concentrations and MAC risk was found in most subgroups (p < 0.05; Fig. 4A), with a stronger correlation in females and CKD 3–4 patients (p for interaction = 0.028; Fig. 4A). Only subgroups < 60 years, male sex, HbA1c ≥ 8.0%, hs-CRP 3.09–32.94 mg/dL, and LCI ≥ 14.66 showed significant positive correlations (p < 0.05; Fig. 4B) between phosphorus concentrations and the risk of developing DF, whereas the remaining subgroups did not have significant correlations between those two variables. Moreover, there was an interaction effect of sex on the risk of developing DF (p for interaction = 0.025; Fig. 4B). Additionally, we conducted three sensitivity analyses (Fig. 5), and the results were essentially similar to those presented in Table 2.

### The role of phosphorus in the diagnosis of MAC and the DF

**Fig. 6:**
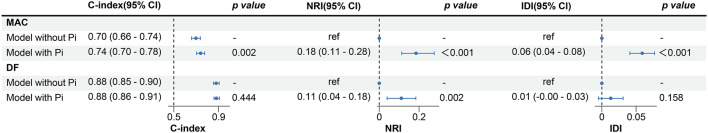
Evaluation of modeling improvement with and without phosphorus for diagnostic prediction of MAC and DF. Model without Pi, including sex, age, HBP history, neuropathy status, the eGFR, HbA1c, and ALB, UA, LDL-C, TC, TG, HDL-C, SOD, hs-CRP, and Ca concentrations. *Ref* reference, *MAC* medial arterial calcification, *DF* diabetic foot, *Pi* phosphorus, *C index* Harrell’s concordance statistic, *NRI* net reclassification improvement, *IDI* integrated discrimination improvement, *DF* diabetes foot, *MAC* medial arterial calcification

The added value of phosphorus for the accuracy of MAC and DF diagnosis in the lower limbs of people with T2DM was compared (Fig. 6). After the addition of phosphorus, the C index of the model for the diagnosis of MAC improved significantly (from 0.70 to 0.74, p < 0.05; Fig. 6), as did its discriminatory power and risk reclassification (IDI 0.06, 95% CI 0.04–0.08; NRI 0.18, 95% CI 0.11–0.28; Fig. 6). In diagnosing DF, the contribution of phosphorus to enhancing diagnostic accuracy was relatively trivial. We further compared the incremental diagnostic value of calcium and the calcium‒phosphorus product for diagnosing MAC and DF (Additional File 1: Table S3). Calcium significantly improved the NRI for MAC (NRI: 0.027, 95% CI 0.001–0.052, p = 0.041) but did not significantly improve the NRI for DF. The calcium‒phosphorus product significantly increased the C index, NRI, and IDI for diagnosing MAC (all p < 0.001), whereas for diagnosing DF, only the IDI improved (IDI 0.010, 95% CI 0.003–0.017, p = 0.008).

## Discussion

In this study, we demonstrated that higher blood phosphorus levels are significantly associated with the risk of developing both MAC and DF in individuals with diabetes. Notably, this association was more pronounced in specific populations. There is also a certain correlation between MAC and DF status. The incorporation of phosphorus concentrations in the diagnosis of MAC in diabetic patients may improve risk categorization.

Phosphorus plays a crucial role in the formation of vascular calcification. It induces the conversion of vascular smooth muscle cells (VSMCs) from a contractile phenotype to an osteochondral phenotype, promotes VSMC matrix calcification, induces apoptosis in VSMCs, inhibits monocyte/macrophage differentiation into osteoclast-like cells, and mediates the regulation of the FGF 23-klotho axis [20, 21]. The diabetic state itself is a contributing factor to vascular calcification [22]. In hyperglycemic states, increased levels of advanced glycation end products (AGEs) bind to their receptors, inducing phenotypic transformation of VSMCs to osteoblasts or promoting the release of matrix vesicles by apoptosis through the CML/RAGE axis, thus accelerating calcium and phosphorus deposition in the vascular wall [23].

Despite generally average phosphorus concentrations, higher phosphorus levels are related to the incidence and extent of vascular calcification in dialysis patients, those with CKD stages 3–4, and younger individuals [4, 10, 11, 24]. In contrast, some studies, such as that by M. H. Grønhøj et al., reported no significant association between coronary artery calcification and blood phosphorus in a middle-aged population without cardiovascular disease or diabetes [25]. In contrast, Alexande et al. reported a negative correlation between coronary artery calcification and serum phosphorus concentrations [26]. The differences in the conclusions may be due to variations in the target population, sample size, adjusted confounding factors, and statistical strategies. Although some publications hold a different view, there are still some indications that phosphorus can predict the development of arterial calcification as far as the current study is concerned [27]. In our study, most of the phosphorus levels were within the normal range. Nevertheless, we found a significant correlation between higher phosphorus levels and the incidence of lower-limb MAC, which was almost linear after adjusting for confounders such as age, sex, HBP history, and neuropathy status. Even with normophosphatemia, greater phosphate concentrations are associated with the development of vascular stiffness and impaired glucose and lipid metabolism, leading to pathological consequences such as T2DM, obesity, and metabolic syndrome [28]. Subgroup and interaction analyses revealed that the influence of phosphorus concentrations on calcification was more significant in females and patients with advanced renal function staging, which is consistent with previous research by Robert N et al. [11]. Calciphylaxis also includes female sex as a risk variable [29]. Differences in estrogen levels and bone structure between men and women may influence calcium and phosphorus metabolism and vascular health [30].

Elevated phosphorus levels are associated with increased mortality and decreased amputation-free survival in patients with critical limb ischemia [31]. Simeone Andrulli et al. reported a significant increase in the risk of developing foot ulcers with increased phosphorus levels in dialysis patients [13]. However, Kaminski et al. reported no significant difference in phosphorus levels between those with and without foot ulcers [32]. Our study revealed that phosphorus appeared to be negatively related to DF development without correcting for confounding factors, but this correlation changed when adjustments were made, reflecting the complex mechanisms of DF. Notably, our subgroup analysis revealed that men are more prone to developing DF, possibly because high phosphate exacerbates conditions such as DF, especially in males at greater risk for metabolic syndrome [33].

Several studies have confirmed that lower-extremity MAC is an independent risk factor for the development of foot ulcers in patients with diabetes and may significantly increase the risk of experiencing future amputations by decreasing vascular compliance and delaying vascular function repair [34–40]. In our analysis, the proportion of patients with comorbid MAC and DF increased with increasing phosphorus level, and the probability of both was positively associated (r = 0.545, p < 0.001; Fig. 3D). Threshold analysis was used to estimate the inflection points for phosphorus-induced MAC and DF (MAC: 3.28 mg/dL; DF: 3.26 and 3.81 mg/dL; Table 3). This discovery has clinical importance for assisting in decision-making and clinical counseling for lower-extremity vascular disease. The RCS plot from a prior study also revealed that phosphorus levels higher than 3.9 mg/dL were linked to a higher risk of coronary artery calcification in young, healthy adults [11]. Controlling blood phosphorus within a specific range may reduce vascular calcification and adverse effects. Noncalcium-based phosphorus binders have been shown to minimize vascular calcification and mortality in dialysis patients [41, 42]. Additionally, although the mediation analysis results of our study indicate that MAC is not a significant mediator in this process (p > 0.05). These findings suggest that the influence of phosphorus concentrations on the risk of developing DF might occur through other pathways, particularly through complex interactions with vascular metabolism [33], which may obscure the role of MAC.

Our study also indicated that adding phosphorus to the diagnostic model enhanced MAC diagnosis. Although phosphorus has been highlighted in numerous studies, research on its effects on vascular disease is currently lacking. For example, the 2017 China DIA-LEDA study did not incorporate blood phosphorus in its risk prediction model for early-onset lower-limb artery disease in patients with diabete [43]. Our study revealed that, in addition to phosphorus, both calcium concentrations and the calcium‒phosphorus product contributed to varying degrees of improvement in the diagnostic models for MAC and DF. These findings underscore the necessity for further mechanistic studies on calcium‒phosphorus metabolism and its role in the development and progression of calcification and DF.

Several strengths of this investigation should be noted. First, strong correlations were identified between phosphorus concentrations and the risk of developing DF and MAC, emphasizing the potential predictive power of blood phosphorus concentrations in identifying high-risk patients. We established a comprehensive target for blood phosphorus regulation by clarifying the phosphorus threshold concerning DF and MAC risk. Subgroup and interaction analyses supported our results, indicating stronger associations between increased phosphorus levels and the risk of developing DF and MAC within specific populations. Additionally, this study provides valuable guidance for blood phosphorus control targets specifically tailored for Chinese patients with diabetes, potentially improving clinical outcomes and preventing the progression of diabetic complications.

However, several limitations must be considered. As this study is a retrospective, cross-sectional analysis, determining causality in the relationships of phosphorus concentrations with the risk of developing MAC and DF is not possible. As this was a single-center study, caution is needed when extrapolating the findings. Potential confounders, such as hemoglobin levels, glucose-lowering treatments, environmental factors, family history, parathyrin, and the ankle–brachial index, may have influenced the outcomes. Additionally, our laboratory does not routinely measure magnesium, FGF-23 and klotho protein levels, which could be relevant to phosphorus metabolism and vascular calcification. The lack of consideration of osteo-metabolic conditions, particularly osteoporosis, due to incomplete clinical records is another limitation. Finally, the study included only diabetic individuals and lacked control data from nondiabetic individuals, making it impossible to verify whether the correlation between phosphorus concentrations and vascular calcification differed between these groups.

## Conclusion

This study demonstrated that elevated phosphorus levels are correlated with the development of lower-extremity MAC and DF in patients with diabetes. These findings highlight the need for prospective studies to further investigate the role of phosphorus concentrations in the risk of developing MAC and DF and to explore potential therapeutic interventions. Additionally, incorporating blood phosphorus measurements into routine diagnostic practices could facilitate the early detection and identification of lower-extremity arterial lesions.

### Supplementary Information

Additional file1 (DOCX 194 kb)

## Data Availability

The datasets analyzed during the current study are not publicly available due to patient privacy but are available from the corresponding author upon reasonable request.

## Abbreviations

MAC: Medial arterial calcification
ESRD: End-stage renal disease
eGFR: Estimated glomerular filtration rate
T2DM: Type 2 diabetes
DF: Diabetes foot
RCS: Restricted cubic spline plot
C index: Harrell’s concordance statistic
IDI: The integrated discriminant index
NRI: Net reclassification index
TyG: Triglyceride-glucose index
LCI: Lipid composite index
CKD: Chronic kidney disease
CKD-EPI: Chronic kidney disease-epidemiology collaboration
VSMCs: Vascular smooth muscle cells
ALB: Albumin
UA: Uric acid
Scr: Creatinine
Pi: Phosphorus
Ca: Corrected calcium
TC: Total cholesterol
TG: Triglycerides
HDL: High-density lipoproteins
LDL-C: Low-density lipoproteins
hs-CRP: Ultrasensitive C-reactive protein
SOD: Superoxide dismutase
HbA1c: Glycosylated hemoglobin
FPG: Fasting plasma glucose
CI: Confidence interval
OR: Odd ratio
CMH: Cochran–Mantel–Haenszel
PSM: Propensity score matching
