# Supplementary material for: Associations of phosphorus concentrations with medial arterial calcification in lower-extremity arteries and diabetic foot in people with diabetes: a retrospective cross-sectional study
Source: Cardiovasc Diabetol. 2024 Jul 25;23:275. doi: 10.1186/s12933-024-02361-5 (PMC11282733; doi:10.1186/s12933-024-02361-5)
Supplement: Supplementary file 1 — Additional file1 (DOCX 194 kb) [file 12933_2024_2361_MOESM1_ESM.docx]

**Additional File 1**


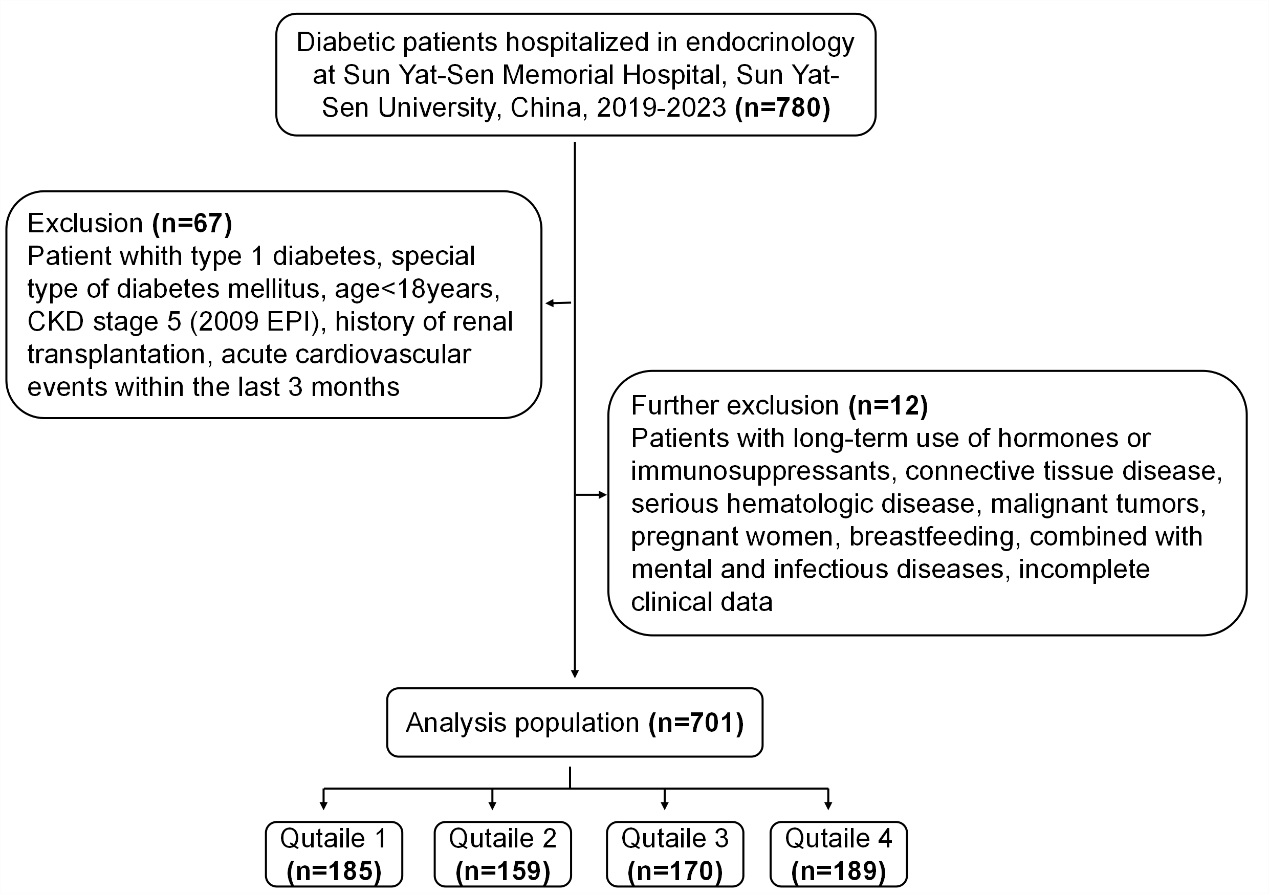


**Figure S1. Flow chart of the study participant selection process**

**Table S1. Baseline characteristics of the MAC, non-MAC, DF and non-DF groups**

|  |  | | Total  (N=701) | Total patients (n=701) | | | | | | |
| --- | --- | --- | --- | --- | --- | --- | --- | --- | --- | --- |
|  |  | |  | non-MAC　(n=368) | MAC  (n=333) | p value |  | non-DF  (n=372) | DF  (n=329) | p value |
| Clinical materials | | |  |  |  |  |  |  |  |  |
|  | Age (years) | | 63.90 (13.29) | 61.04 (13.62) | 67.06 (12.19) | <0.001 |  | 60.6 (13.39) | 67.6 (12.19) | <0.001 |
|  | Sex | |  |  |  | 0.006 |  |  |  | 0.264 |
|  |  | Female (%) | 300 (42.80) | 139 (37.77) | 161 (48.35) |  |  | 167 (44.89) | 133 (40.43%) |  |
|  |  | Male (%) | 401 (57.20) | 229 (62.23) | 172 (51.65) |  |  | 205 (55.11) | 196 (59.57) |  |
|  | BMI (kg/㎡) | | 23.85 (3.85) | 24.04 (3.77) | 23.63 (3.94) | 0.166 |  | 24.68 (3.87) | 22.90 (3.62) | <0.001 |
|  | SBP (mmHg) | | 136.03 (21.58) | 136.98 (21.31) | 136.09 (21.91) | 0.944 |  | 134.85 (19.99) | 137.36 (23.21) | 0.128 |
|  | DBP (mmHg) | | 74.35 (11.18) | 75.74 (11.22) | 72.83 (10.96) | <0.001 |  | 75.27 (11.17) | 73.32 (11.12) | 0.021 |
|  | MAP (mmHg) | | 94.91 (12.54) | 95.81 (12.64) | 93.91 (12.37) | 0.045 |  | 95.13 (12.18) | 94.67 (12.95) | 0.626 |
|  | Hypertension (%) | |  |  |  | 0.007 |  |  |  | <0.001 |
|  |  | No | 271 (38.66) | 160 (43.48) | 111 (33.33) |  |  | 168 (45.16) | 103 (31.31) |  |
|  |  | Yes | 430 (61.34) | 208 (56.52) | 222 (66.67) |  |  | 204 (54.84) | 226 (68.69) |  |
|  | Nephropathy (%) | |  |  |  | 0.005 |  |  |  | <0.001 |
|  |  | No | 379 (54.07) | 218 (59.24) | 161 (48.35) |  |  | 257 (69.09) | 122 (37.08) |  |
|  |  | Yes | 322 (45.93) | 150 (40.76) | 172 (51.65) |  |  | 115 (30.91) | 207 (62.92) |  |
|  | Neuropathy (%) | |  |  |  | <0.001 |  |  |  | <0.001 |
|  |  | No | 223 (31.81) | 138 (37.50) | 85 (25.53) |  |  | 154 (41.40) | 69 (20.97) |  |
|  |  | Yes | 478 (68.18) | 230 (62.50) | 248 (74.47) |  |  | 218 (58.60) | 260 (79.03) |  |
|  | Diabetic Foot (%) | |  |  |  | <0.001 |  |  |  | - |
|  |  | No | 372 (53.07) | 226 (61.41) | 146 (43.84) |  |  | - | - |  |
|  |  | Yes | 329 (46.93) | 142 (38.59) | 187 (56.16) |  |  | - | - |  |
|  | MAC (%) | |  |  |  | - |  |  |  | <0.001 |
|  |  | No | 368 (52.50) | - | - |  |  | 226 (60.75) | 142 (43.16) |  |
|  |  | Yes | 333 (47.50) | - | - |  |  | 146 (39.25) | 187 (56.84) |  |
| Laboratory examination | | |  |  |  |  |  |  |  |  |
|  | Pi (mg/dl) | | 3.55 (0.70) | 3.41 (0.66) | 3.72 (0.70) | <0.001 |  | 3.61 (0.67) | 3.49 (0.73) | 0.025 |
|  | Ca (mg/dl) | | 9.38 (0.46) | 9.34 (0.47) | 9.43 (0.43) | 0.013 |  | 9.53 (0.67) | 10.16 (0.64) | <0.001 |
|  | Ca×Pi (mg/dl)2 | | 33.42 (7.08) | 31.89 (6.66) | 35.12 (7.15) | <0.001 |  | 34.43 (6.97) | 35.44 (7.64) | 0.071 |
|  | ALB (g/L) | | 34.48 (6.95) | 35.43 (7.33) | 33.42 (6.34) | <0.001 |  | 37.98 (5.62) | 30.52 (6.13) | <0.001 |
|  | Scr (μmol/L) | | 94.03 (40.75) | 89.77 (36.57) | 98.73 (44.49) | 0.004 |  | 86.72 (34.94) | 102.29 (45.09) | <0.001 |
|  | eGFR (mL/min/1.73m2) | | 74.81 (25.71) | 79.87 (24.59) | 69.22 (25.80) | <0.001 |  | 80.95 (23.36) | 67.87 (26.50) | <0.001 |
|  | CKD stage (%) | |  |  |  | <0.001 |  |  |  | <0.001 |
|  |  | CKD 1 | 226 (32.24) | 147 (39.95) | 79 (23.72) |  |  | 147 (39.52) | 79 (24.01) |  |
|  |  | CKD 2 | 271 (38.75) | 137 (37.23) | 134 (40.24) |  |  | 154 (41.40) | 117 (35.56) |  |
|  |  | CKD 3 | 170 (24.25) | 71 (19.29) | 99 (29.73) |  |  | 59 (15.86) | 111 (33.74) |  |
|  |  | CKD 4 | 34 (4.85) | 13 (3.53) | 21 (6.31) |  |  | 12 (3.23) | 22 (6.69) |  |
|  | UA (μmol/L) | | 342.55 (118.73) | 341.63 (117.83) | 343.57 (119.85) | 0.829 |  | 368.41 (112.70) | 313.31 (118.76) | <0.001 |
|  | FPG (mmol/L) | | 7.21 (2.70) | 7.28 (2.73) | 7.12 (2.67) | 0.436 |  | 7.02 (2.44) | 7.41 (2.97) | 0.060 |
|  | HbA1c (%) | | 8.78 (2.34) | 9.10 (2.51) | 8.44 (2.08) | <0.001 |  | 9.01 (2.37) | 8.53 (2.28) | 0..007 |
|  | CHOL (mmol/L) | | 4.46 (1.42) | 4.60 (1.44) | 4.31 (1.39) | 0.008 |  | 4.92 (1.42) | 3.94 (1.23) | <0.001 |
|  | TG (mmol/L) | | 1.55 (1.35) | 1.70 (1.65) | 1.38 (0.89) | 0.002 |  | 1.76 (1.69) | 1.31 (0.75) | <0.001 |
|  | LDL-C (mmol/L) | | 2.80 (1.00) | 2.92 (1.02) | 2.68 (0.97) | 0.002 |  | 3.10 (1.03) | 2.46 (0.86) | <0.001 |
|  | HDL (mmol/L) | | 1.04 (0.40) | 1.05 (0.44) | 1.02 (0.36) | 0.446 |  | 1.15 (0.45) | 0.91 (0.29) | <0.001 |
|  | hs-CRP (mg/L) | | 29.41 (47.38) | 25.86 (47.20) | 33.34 (47.33) | 0.037 |  | 8.64 (22.62) | 52.90 (56.29) | <0.001 |
|  | SOD (U/mL) | | 143.26 (34.16) | 148.60 (33.91) | 137.36 (33.50) | <0.001 |  | 155.05 (32.85) | 129.92 (30.54) | <0.001 |

The data are presented as the means (SDs), numbers (percentages), or medians (IQRs). MAC, medial arterial calcification. DF, diabetic foot. Pi, phosphorus. Ca, corrected calcium (mg/dL). Ca×Pi, calcium‒phosphorus product. BMI, body mass index. ALB, albumin. SCr, serum creatinine. eGFR, estimated glomerular filtration rate. UA, uric acid. HbA1c, blood glycosylated hemoglobin. FPG, fasting plasma glucose. CHOL, total cholesterol. TG, triglyceride. LDL-C, low-density lipoprotein cholesterol. HDL-C, high-density lipoprotein cholesterol. LCI, lipid comprehensive index. hs-CRP, high-sensitivity C-reactive protein. SOD, superoxide dismutase. TyG index, triglyceride–glucose index.

**Table S2 Multivariate regression analysis for the risk of developing MAC and DF**

|  |  | | MAC | p value |  | DF | p value |
| --- | --- | --- | --- | --- | --- | --- | --- |
|  | Age (per 10years) | | 1.03 (1.01-1.05) | ＜0.001 |  | 1.28 (1.07-1.54) | 0.008 |
|  | Sex | |  |  |  |  |  |
|  |  | Female (%) | ref | 0.033 |  | ref | 0.188 |
|  |  | Male (%) | 0.68 (0.47-0.97) |  |  | 1.35 (0.86-2.11) |  |
|  | Hypertension (%) | |  |  |  |  |  |
|  |  | No | ref | 0.627 |  | ref | 0.343 |
|  |  | Yes | 0.91 (0.62-1.33) |  |  | 1.25 (0.79-1.98) |  |
|  | Neuropathy (%) | |  |  |  |  |  |
|  |  | No | ref | 0.044 |  | ref | ＜0.001 |
|  |  | Yes | 1.45 (1.01-2.09) |  |  | 2.38 (1.52-3.73) |  |
|  | Pi (mg/dl) | | 2.72 (2.01-3.67) | ＜0.001 |  | 1.51 (1.06-2.13) | 0.021 |
|  | Ca (mg/dl) | | 1.14 (0.76-1.69) | 0.530 |  | 1.36 (0.86-2.14) | 0.192 |
|  | ALB (g/L) | | 0.99 (0.94-1.04) | 0.706 |  | 0.91 (0.85-0.97) | 0.005 |
|  | eGFR (mL/min/1.73m^2^) | | 1.00 (0.99-1.01) | 0.572 |  | 0.99 (0.98-1.00) | 0.012 |
|  | UA (μmol/L) | | 1.00 (1.00-1.00) | 0.883 |  | 1.00 (0.99-1.00) | ＜0.001 |
|  | HbA1c (%) | | 0.91 (0.84-0.98) | 0.015 |  | 0.89 (0.81-0.98) | 0.015 |
|  | CHOL (mmol/L) | | 2.40 (1.11-5.20) | 0.026 |  | 1.23 (0.68-2.23) | 0.502 |
|  | TG (mmol/L) | | 0.78 (0.63-0.96) | 0.022 |  | 0.91 (0.74-1.14) | 0.496 |
|  | LDL-C (mmol/L) | | 0.31 (0.12-0.81) | 0.016 |  | 0.50 (0.18-1.33) | 0.162 |
|  | HDL (mmol/L) | | 0.42 (0.16-1.12) | 0.082 |  | 0.37 (0.13-1.07) | 0.068 |
|  | hs-CRP (mg/L) | | 1.00 (1.00-1.01) | 0.627 |  | 1.02 (1.01-1.03) | ＜0.001 |
|  | SOD (U/mL) | | 1.00 (1.00-1.01) | 0.066 |  | 1.00 (0.99-1.01) | 0.897 |

MAC, medial arterial calcification. DF, diabetic foot. Pi, phosphorus. Ca, corrected calcium (mg/dL). Ca×Pi, calcium‒phosphorus product. ALB, albumin. eGFR, estimated glomerular filtration rate. UA, uric acid. HbA1c, blood glycosylated hemoglobin. CHOL, total cholesterol. TG, triglyceride. LDL-C, low-density lipoprotein cholesterol. HDL-C, high-density lipoprotein cholesterol. LCI, lipid comprehensive index. hs-CRP, high-sensitivity C-reactive protein. SOD, superoxide dismutase.


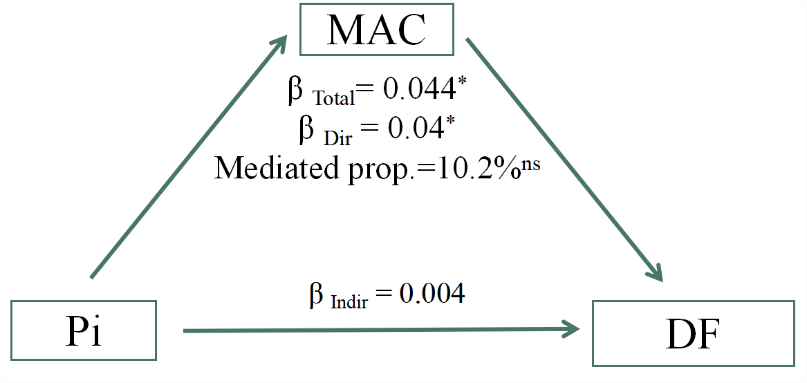


**Figure S2.** Mediating effect of MAC status on the association between Pi concentrations and the risk of developing DF

*, p <0.05. ns, p>0.05. MAC, medial arterial calcification. DF, diabetic foot. Pi, phosphorus

**Table S3. Evaluation of modeling improvement with and without calcium concentrations and Ca-Pi products for the diagnostic prediction of MAC and DF**

|  | **C-index** | |  | **NRI** | |  | **IDI** | |
| --- | --- | --- | --- | --- | --- | --- | --- | --- |
|  | **Index** | **p value** |  | **Index** | **p value** |  | **Index** | **p value** |
| **Ca** |  |  |  |  |  |  |  |  |
| MAC |  |  |  |  |  |  |  |  |
| Model without Ca | 0.738 (0.702-0.775) | - |  | - | - |  | - | - |
| Model with Ca | 0.740 (0.704-0.776) | 0.225 |  | 0.027 (0.001-0.052) | 0.041 |  | 0.001 (-0.001-0.003) | 0.331 |
| DF |  |  |  |  |  |  |  |  |
| Model without Ca | 0.880 (0.855-0.905) |  |  | - | - |  | - | - |
| Model with Ca | 0.881 (0.856-0.906) | 0.440 |  | 0.009 (-0.011-0.028) | 0.375 |  | 0.002 (-0.001-0.005) | 0.190 |
|  |  |  |  |  |  |  |  |  |
| **Ca-Pi product** |  |  |  |  |  |  |  |  |
| MAC |  |  |  |  |  |  |  |  |
| Model without Ca×Pi | 0.687 (0.648-0.726) | - |  | - | - |  | - | - |
| Model with Ca×Pi | 0.740 (0.703-0.776) | <0.001 |  | 0.185 (0.110-0.260) | <0.001 |  | 0.069 (0.050-0.089) | <0.001 |
| DF |  |  |  |  |  |  |  |  |
| Model without Ca×Pi | 0.878 (0.853-0.903) | - |  | - | - |  | - | - |
| Model with Ca×Pi | 0.881 (0.857-0.906) | 0.224 |  | -0.006 (-0.041-0.029) | 0.736 |  | 0.010 (0.003-0.017) | 0.008 |

Model without Ca or Ca×Pi, including sex, age, HBP history, neuropathy status, the eGFR, HbA1c, and ALB, UA, LDL-C, TC, TG, HDL-C, SOD, and hs-CRP concentrations

MAC, medial arterial calcification. DF, diabetic foot. Pi, phosphorus. C index, Harrell's concordance statistic. NRI, net reclassification improvement; IDI, integrated discrimination improvement
